# Attitudinal and Demographic Predictors of Measles-Mumps-Rubella Vaccine (MMR) Uptake during the UK Catch-Up Campaign 2008–09: Cross-Sectional Survey

**DOI:** 10.1371/journal.pone.0019381

**Published:** 2011-05-13

**Authors:** Katrina Brown, Graham Fraser, Mary Ramsay, Ruth Shanley, Noel Cowley, Johan van Wijgerden, Penelope Toff, Michelle Falconer, Michael Hudson, John Green, J. Simon Kroll, Charles Vincent, Nick Sevdalis

**Affiliations:** 1 Department of Surgery and Cancer, Imperial College, London, United Kingdom; 2 Regional Epidemiology Unit, Health Protection Agency, London, United Kingdom; 3 Centre for Infections, Health Protection Agency, London, United Kingdom; 4 NHS Ealing, London, United Kingdom; 5 NHS Brent, London, United Kingdom; 6 NHS Halton & St Helens, Cheshire, United Kingdom; 7 Centre for Emergency Preparedness and Response, Health Protection Agency, Wiltshire, United Kingdom; 8 Central and North-West London NHS Foundation Trust, London, United Kingdom; 9 Department of Medicine, Imperial College, London, United Kingdom; University of Liverpool, United Kingdom

## Abstract

**Background and Objective:**

Continued suboptimal measles-mumps-rubella (MMR) vaccine uptake has re-established measles epidemic risk, prompting a UK catch-up campaign in 2008–09 for children who missed MMR doses at scheduled age. Predictors of vaccine uptake during catch-ups are poorly understood, however evidence from routine schedule uptake suggests demographics and attitudes may be central. This work explored this hypothesis using a robust evidence-based measure.

**Design:**

Cross-sectional self-administered questionnaire with objective behavioural outcome.

**Setting and Participants:**

365 UK parents, whose children were aged 5–18 years and had received <2 MMR doses before the 2008–09 UK catch-up started.

**Main Outcome Measures:**

Parents' attitudes and demographics, parent-reported receipt of invitation to receive catch-up MMR dose(s), and catch-up MMR uptake according to child's medical record (receipt of MMR doses during year 1 of the catch-up).

**Results:**

Perceived social desirability/benefit of MMR uptake (OR = 1.76, 95% CI = 1.09–2.87) and younger child age (OR = 0.78, 95% CI = 0.68–0.89) were the only independent predictors of catch-up MMR uptake in the sample overall. Uptake predictors differed by whether the child had received 0 MMR doses or 1 MMR dose before the catch-up. Receipt of catch-up invitation predicted uptake only in the 0 dose group (OR = 3.45, 95% CI = 1.18–10.05), whilst perceived social desirability/benefit of MMR uptake predicted uptake only in the 1 dose group (OR = 9.61, 95% CI = 2.57–35.97). Attitudes and demographics explained only 28% of MMR uptake in the 0 dose group compared with 61% in the 1 dose group.

**Conclusions:**

Catch-up MMR invitations may effectively move children from 0 to 1 MMR doses (unimmunised to partially immunised), whilst attitudinal interventions highlighting social benefits of MMR may effectively move children from 1 to 2 MMR doses (partially to fully immunised). Older children may be best targeted through school-based programmes. A formal evaluation element should be incorporated into future catch-up campaigns to inform their continuing improvement.

## Introduction

Uptake of measles-mumps-rubella (MMR) vaccine in the UK has remained below 85% for over a decade, and currently only 83% of five year-olds are adequately immunised in line with the recommended two-dose schedule [1]. This suboptimal vaccine coverage leaves the UK population at risk of a measles epidemic [3], [4]. In response to this, an MMR catch-up campaign was launched in 2008 to improve MMR coverage among children who missed MMR doses at scheduled age (dose 1 at ∼13 months, dose 2 at ∼3 years and 4 months). From 1^st^ September 2008 Primary Care Trusts (PCTs) across the UK were instructed to offer catch-up MMR to children aged 13 months to 18 years [1]. Children were prioritised first by MMR doses received, then by age, such that younger children with no MMR doses on their General Practice (GP) or PCT record were the primary targets for the campaign. GPs/PCTs were advised to send postal invitations to parents/caregivers of eligible children asking them to bring their child to the GP surgery for catch-up MMR. Department of Health (DH) trial sentinel data for the first year of the catch-up campaign indicate a 5.1% increase in full MMR coverage among 5–18 year olds and a 2% decrease in the number who have no MMR doses recorded [2].

Catch-up campaigns providing a vaccine to those who missed it at scheduled age (for example, because their parents could not access it or chose to reject it) are typically only moderately successful, immunising less than 50% of their target populations [3]–[6], with only 20–25% uptake in some campaigns [3], [4]. Most campaigns fail either to collect or report relevant evaluation data indicating why parents in their target populations accepted or rejected MMR within the catch-up, however withheld or missing parent consent (whilst some parents actively refuse the vaccine, often a greater number simply fail to respond to the invitation – their consent may be consciously withheld or unconsciously omitted, but either way their children are not immunised by default in the absence of explicit consent) has been implicated in 45–62% of cases where an eligible child has not received catch-up MMR within school-based programmes [5], [7]. A number of attitudinal and demographic factors have been linked with MMR uptake within the routine schedule (see below) [8]–[11], and these factors may also relate to catch-up MMR uptake. The present work tests this hypothesis by identifying univariate and multivariate predictors of catch-up MMR uptake during the 2008–09 MMR catch-up campaign.

### Factors related to routine MMR uptake

- Beliefs about and previous experience of MMR safety and efficacy
- Beliefs about severity, susceptibility and possible benefits (e.g. natural immunity) of measles
- Perceived social desirability and value of community benefit of MMR uptake
- Satisfaction with and trust in official (e.g. Department of Health, NHS) and unofficial (e.g. internet and lay advice) information around MMR and measles
- Practical barriers to clinic attendance (e.g. availability of appointments)
- Parent age and socioeconomic status (an inverted U curve is observed, with MMR uptake lower at the extreme ends of both parameters)

## Methods

### Ethics statement

The Health Protection Agency and PCTs involved classified the work as a service evaluation not requiring ethical approval as results were anonymised for analysis. Consent to participate was implied through questionnaire completion.

### Participants

Child Health Information Systems (CHIS) in three UK PCTs (two in London, one in north-west England) were used to identify all children aged 5–17 years and with suboptimal CHIS-recorded MMR status (<2 doses) at 1^st^ September 2008 (the first day of the UK MMR catch-up campaign 2008–09). From this population, 2,300 children were randomly selected with stratification by child age. This sample size provided 80% power for hierarchical multiple regression to detect small to medium effects at the 0.05 significance level with a 20% response rate. PCTs provided postal and telephone contact details for the parent/guardian(s) of each child, plus the child's date of birth and MMR dose history.

### Materials and procedure

**Figure 1:**
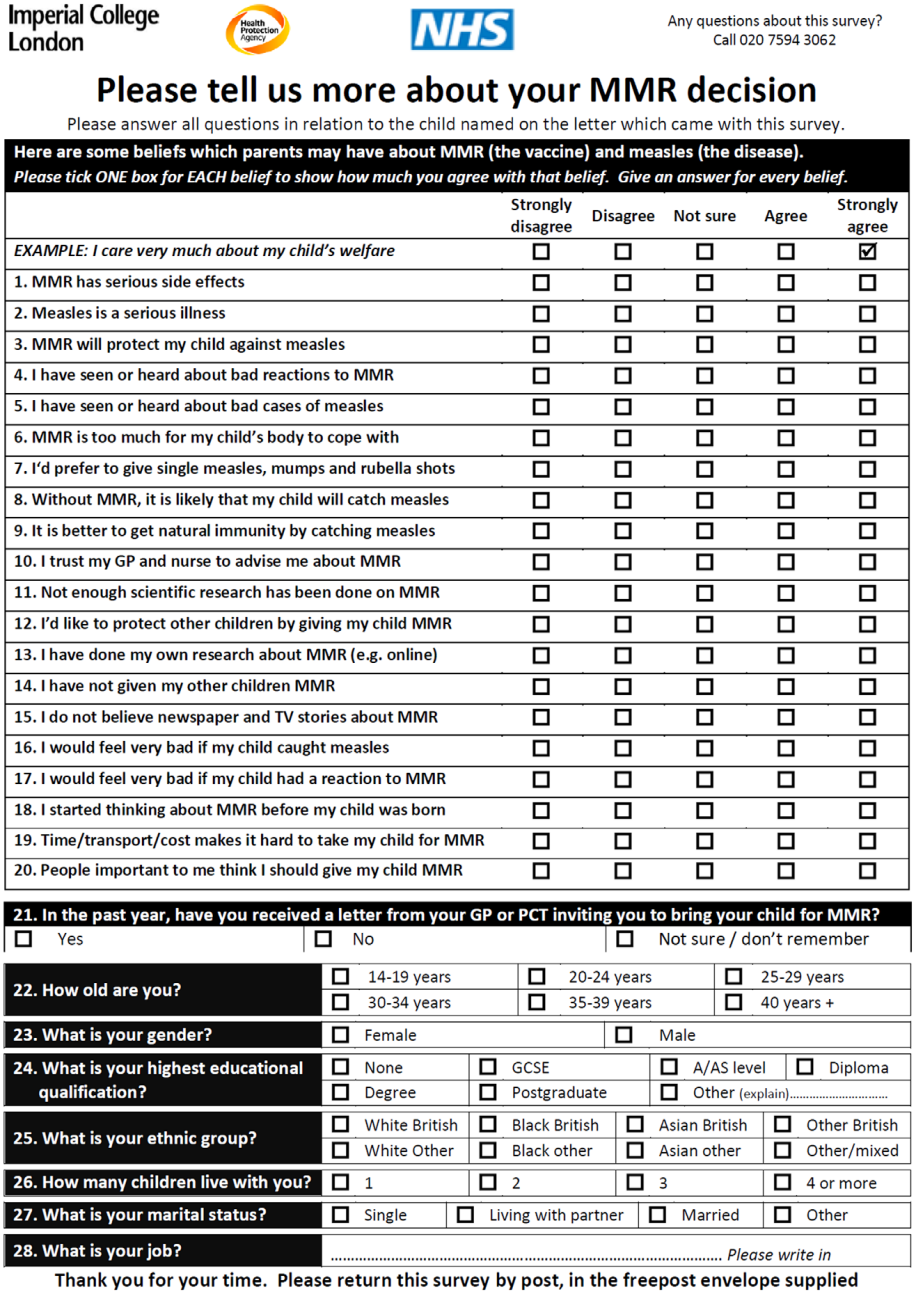
Questionnaire used to assess attitudes, demographics, past behaviour and receipt of MMR catch-up invitation among parents of children eligible to receive catch-up MMR vaccine within the 2008–09 UK MMR catch-up campaign.

The internal consistency, test-retest reliability, concurrent and predictive validity of the questionnaire (Figure 1) has been demonstrated previously [12]. The questionnaire comprised 20 attitude items and seven demographic items all derived from the literature on factors underpinning parents' routine schedule MMR decisions [8]–[11], and a single item assessing self-reported receipt of a postal MMR catch-up invitation. Attitude items took the form of statements with which the respondent indicated their level of agreement on five-point scales (1 = strongly disagree, 5 = strongly agree), for every attitude item a higher score indicated more ‘pro-MMR’ attitude. The attitude items (except item 19 assessing practical barriers) collapsed into four scales with adequate reliability (see Table S1 for details of items in each scale. Cronbach's alphas = 0.59–0.76). Demographic data collected were parent (respondent) age, sex, highest educational qualification, ethnic group, number of children, marital status and job; responses were provided using tick-box options for all but the job item, which was free-text.

Between April and September 2009, a copy of the questionnaire was posted to the parent/guardian of every child in the sample, along with a cover letter explaining the purpose and provenance of the survey, a freepost return envelope, and a notice advising in languages most commonly used in the PCTs that translations were available on request. A maximum of two postal and two telephone reminders were made, at approximately 3–4 and 6–7 weeks after the first copy was sent. Postal reminders contained replacement questionnaires, and telephone reminders comprised an invitation to respond verbally to the questionnaire during the call.

CHIS-recorded receipt of MMR dose(s) during the first year of the catch-up campaign (1^st^ September 2008–31^st^ August 2009), and postcode-level Indices of Multiple Deprivation 2007 data (IMD2007 [13]), were obtained for the entire sample including non-respondents. Where MMR dose history obtained at the end of the studied period differed from that which had been provided at the start of the period, the most up-to-date history was used. Free-text responses to the job item were coded by two independent analysts (very good agreement between analysts: Cohen's Kappa 0.91) to the 8-class version of the National Statistics Socio-Economic Classification (NS-SEC [14]), where code 1 is the highest socio-economic class (higher managerial/higher professional/large employer) and code 8 the lowest (never worked/long-term unemployed/student etc); respondents classifying themselves as ‘mother’, ‘housewife’ or similar were coded to category 8.

### Analysis

Data were analysed using SPSS v 17.0 (SPSS Inc. Chicago, IL). Participation rates and participant characteristics were assessed using all available data on the entire sample. Missing values were imputed using within-participant scale means for scales of 5 items or more where up to 2 items were missing. Scale scores were calculated by summing scores (including imputed values) for individual items comprising the scale then dividing by the number of items in the scale.

All further analyses were completed first for the sample as a whole, then with participants split into two groups: those whose child had received no MMR doses at the start of the catch-up period (henceforth referred to as ‘unimmunised’), and those whose child had received one MMR dose at that time (‘partially immunised’). Univariate (Chi-square tests and ordinal regression for nominal data, Mann-Whitney tests and ANCOVA for ordinal data) and multivariate (hierarchical logistic regression) comparisons were made within each group between those who gave MMR dose(s) during the catch-up period and those who didn't.

## Results

### Response rate and respondent characteristics

365 of 2,300 (15.9%) identified cases returned a completed questionnaire. Achieved power differed minimally from planned power despite the response rate being 4% lower than expected. There was no difference in response rate by MMR status, but respondents had younger children (p<0.01) and lived in less deprived postcode areas (p<0.001) than did non-respondents (Table 1).

**Table 1:** Participation rates and representativeness.

	n	n(%) / Mean(SD)		p
		Participants	Non-participants	
MMR status at end of data collection†				
0 doses	1166	182 (15.6)	984 (84.4)	0.31
1 dose	882	135 (15.3)	747 (84.7)	
2 doses	252	48 (19.0)	204 (81.0)	
Child age (years) at end of data collection‡	-	9.8 (3.6)	10.4 (3.8)	<0.01
IMD2007 score‡	-	26.28 (15.64)	31.43 (17.14)	<0.001
Total	2300	365 (15.9)	1935 (84.1)	-

† : n(%), p values for Chi-square test;

‡ : mean(SD), p values for independent samples t-test.

### Factors associated with catch-up MMR uptake in univariate analyses

See Tables 2 and 3. In the sample as a whole, catch-up MMR uptake was associated with younger child age (p<0.001), perceived social desirability/benefit of MMR uptake (p<0.001, 7% variance), pro-MMR feelings (p<0.001, 4% variance), satisfaction with available official information around MMR (p<0.01, 3% variance), and concern about measles (p<0.05, 1% variance). Less than one-third of parents reported having received an MMR catch-up invitation in the past year, and receipt of an invitation was not associated with catch-up MMR uptake, when child age and deprivation were taken into account. The individual items (see Table S1) explaining the most variance in catch-up MMR uptake for the whole sample were disbelieving serious MMR side effects, valuing community benefit of immunisation, and perceiving peers/family to be pro-MMR (all p<0.001, 5% variance). With the sample split by MMR status at the start of the catch-up campaign (unimmunised versus partially immunised), univariate associations were largely as described above for both groups. However, measles beliefs showed no association with catch-up MMR uptake in these smaller subsamples (p>0.05). In addition, catch-up MMR uptake was linked with younger parent age only among parents of unimmunised children (p<0.05), and with lower educational attainment only among parents of partially immunised children (p<0.01).

**Table 2:** Demographic characteristics by catch-up MMR uptake.

	All cases		Unimmunised		Partially immunised	
	Eligible (n)	Catch-up MMR uptake(n (%))	Eligible (n)	Catch-up MMR uptake(n (%))	Eligible (n)	Catch-up MMR uptake(n (%))
**Child age (years)†**						
5–6	115	44 (38)	69	17 (25)	46	27 (59)
7–8	57	10 (18)	36	5 (14)	21	5 (24)
9–10	52	5 (10)	31	2 (6)	21	3 (14)
11–12	78	6 (8)	40	3 (8)	38	3 (8)
13–14	22	5 (23)	19	2 (11)	13	3 (23)
15–16	12	2 (17)	6	2 (33)	6	0 (0)
17–18	19	0 (0)	12	0 (0)	7	0 (0)
**IMD 2007 score‡**						
<sample mean (31.4)	240	42 (18)	145	22 (15)	95	20 (21)
≥sample mean	123	29 (24)	67	9 (13)	56	20 (36)
**Parent age (years)**						
20–24	8	2 (25)	5	1 (3)	3	1 (3)
25–29	21	5 (24)	13	3 (10)	8	2 (5)
30–34	47	15 (32)	25	6 (20)	22	9 (23)
35–39	99	23 (23)	65	12 (40)	34	11 (28)
40+	179	24 (13)	100	8 (27)	79	16 (41)
**Parent highest qualification**						
None	26	7 (27)	17	2 (12)	9	5 (56)
GCSE/O-level	82	18 (22)	50	9 (18)	32	9 (28)
A/AS-level	45	11 (24)	25	3 (12)	20	8 (40)
Diploma	73	10 (14)	39	3 (8)	34	7 (21)
Degree	74	12 (16)	45	7 (16)	29	5 (17)
Postgraduate degree	40	9 (23)	28	6 (21)	12	3 (25)
Other	4	0 (0)	2	0 (0)	2	0 (0)
**Parent ethnicity**						
White British	248	54 (22)	144	22 (15)	104	32 (31)
Black British	16	2 (13)	10	1 (10)	6	1 (17)
Asian British	24	2 (8)	14	0 (0)	10	2 (20)
Other British	3	1 (33)	2	1 (50)	1	0 (0)
White other	18	5 (28)	12	4 (33)	6	1 (17)
Black other	5	2 (40)	3	1 (33)	2	1 (50)
Asian other	26	3 (12)	18	2 (11)	8	1 (13)
Other or mixed	6	1 (17)	2	0 (0)	4	1 (25)
**Number of children**						
1	56	14 (25)	32	4 (13)	24	10 (42)
2	172	36 (21)	99	17 (17)	73	19 (26)
3	75	10 (13)	42	4 (10)	33	6 (18)
4+	46	10 (22)	30	5 (17)	16	5 (31)
**Parent marital status**						
Single	63	13 (21)	42	7 (17)	21	6 (29)
Cohabiting	58	13 (22)	25	5 (20)	23	8 (35)
Married	222	37 (17)	127	14 (11)	95	23 (24)
Other	25	6 (24)	18	4 (22)	7	2 (29)
**Parent job (NS-SEC)**						
1	39	7 (18)	26	2 (8)	13	5 (38)
2	31	8 (26)	17	4 (24)	14	4 (29)
3	21	7 (33)	11	2 (18)	10	5 (50)
4	12	1 (8)	10	1 (10)	2	0 (0)
5	32	5 (16)	24	3 (13)	8	2 (25)
6	54	7 (13)	30	2 (7)	24	5 (21)
7	49	14 (29)	24	5 (21)	25	9 (36)
8	91	17 (19)	51	9 (18)	40	8 (20)

**Table 3:** Attitudes and catch-up invitation receipt by catch-up MMR uptake.

	All cases			Unimmunised			Partially immunised		
	Mean(SD) / n(%)		Effect size and p for no uptake vs uptake	Mean(SD) / n(%)		Effect size and p for no uptake vs uptake	Mean(SD) / n(%)		Effect size and p for no uptake vs uptake
	No catch-up uptake	Catch-up uptake		No catch-up uptake	Catch-up uptake		No catch-up uptake	Catch-up uptake	
*n*	*281–290*	*65–70*		*152–182*	*27–31*		*98–110*	*27–41*	
MMR beliefs	2.8 (0.7)	3.1 (0.7)	0.04***	2.7 (0.7)	3.0 (0.6)	0.03*	2.9 (0.7)	3.2 (0.7)	0.04*
Measles beliefs	3.8 (0.7)	4.0 (0.6)	0.01*	3.8 (0.7)	3.9 (0.6)	0.01	3.8 (0.6)	4.0 (0.6)	0.01
Social and parenting beliefs	3.2 (1.0)	3.7 (0.8)	0.07***	3.1 (1.1)	3.4 (0.9)	0.02*	3.4 (0.9)	4.0 (0.6)	0.10***
Information source beliefs	3.0 (0.7)	3.2 (0.7)	0.03*	2.9 (0.7)	3.1 (0.8)	0.02*	3.2 (0.6)	3.3 (0.6)	0.03*
Practicalities	4.3 (0.8)	4.4 (0.7)	0.002	4.3 (0.8)	4.3 (0.8)	0.003	4.2 (0.8)	4.6 (0.6)	0.03*
MMR catch-up invitation received in past year	77 (26)	30 (42)	-	54 (30)	15 (48)	-	23 (21)	15 (37)	-

P values and effect size (partial Eta squared) from ANCOVA, adjusted for child age and IMD2007 score.

* = p<.05,

** = p<.01,

*** = p<.001.

### Multivariate predictors of catch-up MMR uptake

See Table 4. In the sample as a whole, catch-up MMR uptake was predicted by perceived social desirability/benefit of MMR uptake (OR = 1.76, 95% CI = 1.09–2.87) and younger child age (OR = 0.78, 95% CI = 0.68–0.89). However, the profile of multivariate predictors differed substantially between parents of previously unimmunised children and parents of previously partially immunised children. In the former, catch-up MMR uptake was predicted only by receipt of catch-up invitation (OR = 3.45, 95% CI = 1.18–10.05), younger parent age (OR = 0.58, 95% CI = 0.36–0.92), and residence in a less deprived postcode (OR = 0.96, 95% CI = 0.92–0.99). In the latter, catch-up MMR uptake was predicted only by perceived social desirability/benefit of MMR uptake (OR = 9.61, 95% CI = 2.57–35.97), lower parent educational attainment (OR = 0.08, 95% CI = 0.01–0.58), and younger child age (OR = 0.44, 95% CI = 0.29–0.66).

**Table 4:** Independent predictors of catch-up MMR uptake.

Predictor	Odds ratios (95% CIs)		
	All cases	Unimmunised	Partially immunised
*n*	*284*	*174*	*110*
*Nagelkerke R^2^*	*0.27*	*0.28*	*0.61*
Child age	0.78 (0.68–0.89)	0.93 (0.79–1.10)	0.44 (0.29–0.66)
IMD2007 score	0.99 (0.96–1.01)	0.96 (0.92–0.99)	0.98 (0.94–1.02)
Parent age	0.79 (0.57–1.10)	0.58 (0.36–0.92)	1.41 (0.63–3.15)
Parent BME ethnicity	0.87 (0.38–1.99)	1.12 (0.33–3.77)	1.31 (0.23–7.33)
Parent married	0.51 (0.25–1.07)	0.41 (0.14–1.16)	0.20 (0.04–1.10)
Parent education≥degree	0.96 (0.43–2.17)	3.21 (0.98–10.47)	0.08 (0.01–0.58)
Number of children	0.98 (0.67–1.44)	1.35 (0.80–2.27)	0.58 (0.29–1.19)
Parent occupation	0.97 (0.83–1.12)	1.14 (0.92–1.41)	0.74 (0.53–1.05)
Catch-up invitation received	1.72 (0.83–3.57)	3.45 (1.18–10.05)	2.00 (0.44–9.09)
MMR beliefs	1.22 (0.64–2.31)	1.61 (0.63–4.11)	0.35 (0.09–1.36)
Measles beliefs	1.01 (0.55–1.86)	1.71 (0.74–3.97)	0.24 (0.05–1.06)
Social and parenting beliefs	1.76 (1.09–2.87)	0.82 (0.41–1.64)	9.61 (2.57–35.97)
Information source beliefs	1.18 (0.59–2.34)	1.34 (0.49–3.67)	5.12 (0.95–27.52)
Practicalities	0.94 (0.57–1.55)	0.76 (0.39–1.49)	1.93 (0.60–6.26)

## Discussion

### Summary of current findings and relation to previous work

Perceiving MMR uptake to be socially desirable/beneficial, and having a younger child were the only independent predictors of MMR uptake during the catch-up campaign. Though univariate analyses indicated that catch-up MMR acceptors differed from catch-up MMR decliners also on MMR beliefs, measles beliefs, and information source beliefs, these factors were not independently responsible for variability in uptake behaviour. Independent predictors of catch-up MMR uptake differed by whether the dose in question was the first the child was to receive or the second. Acceptance of a first dose was primarily predicted by receipt of a catch-up invitation, and no attitudinal factors were implicated in this behaviour. Acceptance of a second dose was predicted most strongly by perceived social consequences of MMR immunisation, and invitation receipt had no bearing on this behaviour.

Whilst to our knowledge attitudinal and demographic predictors of MMR uptake during catch-up campaigns have not previously been modelled in multivariate analyses, the present findings may be usefully compared with the few relevant models predicting routine MMR uptake. Perceived social desirability/benefit of MMR uptake, a key predictor in this work, was unrelated to PCT-recorded routine MMR uptake in 1999–2000 [15], however perceived importance of eradicating rubella (similar to value placed on social benefit of MMR uptake) was a significant predictor of parent-reported MMR uptake in 2003–2004 [16]. Other key predictors in these studies were previous immunisation behaviour, trust in information sources, and belief in MMR side effects, and whilst these factors were related to catch-up MMR uptake in our univariate analyses, their independent impacts on catch-up behaviour were not significant. These differences may reflect evolving views on MMR in society as the MMR controversy abates, or the different ages of children whose parents participated in the routine uptake studies versus our catch-up study. Our univariate findings generally correlate with results from relevant studies of routine MMR uptake [17], [18], with some interesting differences, again perhaps a function of study period or population. For example, in the present work most parents anticipated regret [19] as a consequence both of MMR reaction and of measles infection, and the extent of this regret did not vary by catch-up MMR uptake, however in 2004 [17] routine MMR rejectors were more likely than MMR acceptors to anticipate regret for MMR reaction, and vice-versa. In the same 2004 study [17], benefitting the community by immunising one's own child was one of the few factors on which routine MMR acceptors and rejectors did not differ, whilst in the present work this was one of the most polarising issues. In the only post-MMR controversy assessment of attitudinal factors underpinning catch-up MMR uptake (during the London 2004/5 primary school campaign) [3], MMR safety concerns (particularly autism) were the most frequently cited reasons for catch-up MMR rejection. In our multivariate analyses, however, these factors did not figure, again perhaps a function of time elapsing since the controversy [20], [21] and parents of older children being questioned.

### Implications for policy, practice and further research

There are at least three possible explanations for the finding that attitudes, particularly those about the social aspects of MMR immunisation, were more predictive of uptake among parents who were to give a second dose of MMR than they were among parents who were to give a first dose: parents deciding about a second dose (a) had chosen not to give that second dose previously but the catch-up campaign changed their minds; (b) had always held ‘pro-MMR’ beliefs but had simply forgotten to obtain that second dose and the campaign reminded them; or (c) were more able to consider ‘peripheral’ factors like social benefits and norms since they were reassured about MMR risks following their child's earlier receipt of an MMR dose. These explanations require further investigation, perhaps most effectively with a qualitative methodology, but they offer some useful directions for future catch-up programmes or interventions within the routine schedule. This work also indicates that the attitudinal and demographic profile of parents who immunise during a catch-up campaign is different to that of parents who immunise within the routine schedule: key predictors of routine MMR receipt in this population are being of black/minority ethnicity and having positive MMR beliefs [12], but those factors did not figure in the prediction of catch-up MMR receipt. Catch-up campaigns may therefore require different information materials, health professional approaches, and population targeting than do routine campaigns. Finally, the work demonstrates a clear relationship between younger child age and catch-up MMR receipt in the context of this PCT-based programme. School-based approaches may be more effective in reaching older children [6].

It seems viable and desirable on the basis of the present findings to roll out the measurement instrument with a modified administration method in advance of the next catch-up campaign. This would allow collection of baseline attitudinal data, which can then be compared to post-campaign attitudes aiming to ascertain campaign efficacy in improving attitudes and beliefs. This strategy may be implemented over a large number of PCTs in a nationwide catch-up programme, or over individual PCTs running local programmes; the data can then be combined using meta-analytic techniques to obtain a comprehensive and reliable picture of predictors of MMR receipt during catch-up initiatives, thus contributing directly to rendering such campaigns more amenable to formal evaluation.

### Strengths and limitations

This study is one of only a handful to explore factors underpinning catch-up MMR uptake [5], [7]. Despite the persistent disappointing performance [3]–[6] of catch-up immunisation campaigns, evaluation to date has been sparse and methodologically limited. The present work used a psychometrically robust, evidence-based instrument [12] to assess a broad spectrum of predictors of MMR uptake, with a demographically diverse sample of catch-up MMR acceptors and rejectors, and an objective outcome measure. These methodological strengths are uncommon even in the much larger literature on routine schedule MMR decision-making [9]. Importantly, these methodological advances allowed univariate and multivariate analyses which are, to our knowledge, unique contributions to the catch-up MMR uptake prediction knowledge base. Further, the work demonstrates the viability of evaluating future catch-up campaigns with the instrument used here. However, the work is not without limitations. Though the analysis was adequately powered for statistical comparisons between those who did and did not accept catch-up MMR within the sample, the modest response rate may have compromised the generalisability of the sample to the wider population from which it was drawn. The response rate was lower than has been obtained previously in catch-up and routine MMR populations [5], [7], [17], [18] – likely due in part to poor PCT data quality [22] inflating the denominator in our participation rate calculations (previous catch-up studies obtained more reliable denominators by sampling through schools or from subpopulations of parents who had already responded to an immunisation consent request) – however our study is comparable to those with higher response rates with regard to ratio of MMR-acceptors to MMR-rejectors, and sample demographics, thus the differences we note above between predictors of catch-up and routine MMR uptake are unlikely to be explained by response rate or respondent characteristics alone. Though efforts were made to facilitate participation among hard-to-reach groups [23], [24], and the sample was reasonably varied in educational attainment, occupation and ethnicity, those deprived, low literacy, non English-speaking populations who fail both to respond to questionnaires about immunisation and to attend for immunisation are perhaps not as well-represented here as their wealthier, more literate counterparts [8], [9], [25]. We chose to assess the relationship between MMR invitation and MMR uptake via parent report of receipt rather than PCT/GP record of sending, because we sought to assess the impact of the invitation on the recipient, not the quality or success of PCT/GP efforts to send the invitation out; however, parent report is open to recall bias (parents may have received their invitation 6–12 months before they completed our questionnaire, and simply forgot about the invitation in this period) and experimenter bias (parents may have denied receiving an invitation in order to justify not obtaining MMR for their child). Some evidence suggests that receipt of immunisation invitation letters will be forgotten or denied by around 50% of parents [26], therefore our data may underestimate the number of parents who received invitations and thus overestimate the effect of invitation receipt on MMR uptake. However, to the extent that an invitation is only as useful as it is memorable or noticeable, novel invitation formats (for example, a personalised ‘birthday card’ to be displayed rather than a standard letter to be read and discarded) may have more of an effect on uptake [22]. Finally, the cross-sectional design of the study means it is not possible to ascertain causality: we cannot infer whether positive attitudes and MMR invitation receipt caused catch-up MMR uptake, or whether catch-up uptake created more positive attitudes and heightened parents' awareness of/memory for having received an invitation.

### Conclusion

Receipt of a first-ever MMR dose during the catch-up period was predicted most strongly by receipt of an invitation letter from the GP/PCT, whilst receipt of a second dose during the campaign was predicted most strongly by appreciation of the social benefits (for oneself and for the community) of accepting MMR. Future local and national catch-up programmes should be designed with these differential motivations in mind, and can be robustly evaluated using the attitude assessment tool employed here.

## Supporting Information

Table S1 Individual attitudes items by catch-up MMR uptake.
(DOC)
