# Supplementary material for: Attitudinal and Demographic Predictors of Measles-Mumps-Rubella Vaccine (MMR) Uptake during the UK Catch-Up Campaign 2008–09: Cross-Sectional Survey
Source: PLoS One. 2011 May 13;6(5):e19381. doi: 10.1371/journal.pone.0019381 (PMC3094347; doi:10.1371/journal.pone.0019381)
Supplement: Table S1 — Individual attitudes items by catch-up MMR uptake. (DOC) [file pone.0019381.s001.doc]

**Supplementary Table 1: Individual attitudes items by catch-up MMR uptake**

|  | **All cases** | | | **Unimmunised** | | | **Partially immunised** | | |
| --- | --- | --- | --- | --- | --- | --- | --- | --- | --- |
|  | **Mean(SD) / n(%)** | | **Effect size and p for no uptake vs uptake** | **Mean(SD) / n(%)** | | **Effect size and p for no uptake vs uptake** | **Mean(SD) / n(%)** | | **Effect size and p for no uptake vs uptake** |
|  | **No catch-up uptake** | **Catch-up uptake** |  | **No catch-up uptake** | **Catch-up uptake** |  | **No catch-up uptake** | **Catch-up uptake** |  |
| *n* |  |  |  | *152-182* | *27-31* |  | *98-110* | *27-41* |  |
| MMR side effects ^a^ | 2.8 (1.1) | 3.3 (1.0) | 0.05*** | 2.7 (1.1) | 3.0 (0.9) | 0.02 | 2.9 (1.1) | 3.6 (1.0) | 0.06** |
| MMR effectiveness ^a^ | 4.0 (0.9) | 4.2 (0.8) | 0.01 | 4.0 (0.9) | 4.2 (0.5) | 0.01 | 4.1 (0.8) | 4.2 (0.9) | 0.004 |
| MMR reaction experience ^a^ | 2.3 (1.1) | 2.6 (1.2) | 0.02* | 2.3 (1.2) | 2.5 (1.2) | 0.004 | 2.2 (1.0) | 2.7 (1.1) | 0.05** |
| Immune overload ^a^ | 3.1 (1.2) | 3.4 (1.2) | 0.03* | 3.0 (1.3) | 3.2 (1.1) | 0.01 | 3.2 (1.0) | 3.6 (1.2) | 0.04* |
| Preference for separate shots ^a^ | 2.5 (1.3) | 2.8 (1.3) | 0.02** | 2.4 (1.3) | 2.5 (1.3) | 0.01 | 2.8 (1.2) | 3.1 (1.2) | 0.01 |
| MMR uptake for siblings ^a^ | 3.4 (1.4) | 3.9 (1.2) | 0.03** | 3.3 (1.5) | 3.9 (1.1) | 0.03* | 3.2 (0.9) | 3.2 (0.9) | 0.01 |
| Anticipated regret MMR reaction ^a^ | 1.6 (0.8) | 1.7 (0.9) | 0.002 | 1.5 (0.8) | 1.7 (1.0) | 0.01 | 3.4 (0.2) | 3.2 (1.3) | 0.001 |
| Measles severity ^b^ | 4.2 (0.9) | 4.4 (0.7) | 0.003 | 4.2 (0.9) | 4.3 (0.6) | 0.001 | 4.3 (0.8) | 4.5 (0.8) | 0.01 |
| Measles experience ^b^ | 3.6 (1.0) | 3.8 (1.1) | 0.01 | 3.6 (1.1) | 4.1 (1.0) | 0.02* | 3.6 (1.0) | 3.6 (1.2) | 0.003 |
| Measles susceptibility ^b^ | 3.3 (1.1) | 3.6 (0.9) | 0.02** | 3.3 (1.1) | 3.6 (0.9) | 0.01 | 3.4 (1.0) | 3.7 (0.8) | 0.02 |
| Natural immunity ^b^ | 3.6 (1.0) | 3.8 (0.9) | 0.01 | 3.6 (1.0) | 3.8 (0.9) | 0.003 | 3.6 (0.9) | 3.9 (0.8) | 0.02 |
| Anticipated regret measles ^b^ | 4.1 (1.0) | 4.1 (1.0) | 0.001 | 4.1 (1.0) | 3.9 (1.1) | 0.004 | 1.7 (0.8) | 1.6 (0.7) | 0.003 |
| Protecting community ^c^ | 3.4 (1.1) | 3.9 (0.9) | 0.05*** | 3.3 (1.2) | 3.5 (1.0) | 0.01 | 2.7 (1.1) | 3.0 (1.3) | 0.09*** |
| Pro-MMR social norms ^c^ | 3.0 (1.2) | 3.5 (1.1) | 0.05*** | 2.9 (1.2) | 3.2 (1.1) | 0.02 | 3.2 (1.2) | 3.8 (1.0) | 0.07** |
| Trust GP/nurse MMR advice ^d^ | 3.5 (1.1) | 3.8 (0.9) | 0.03** | 3.5 (1.2) | 3.7 (0.9) | 0.02 | 2.9 (1.0) | 3.1 (1.0) | 0.03* |
| MMR scientific research ^d^ | 2.7 (1.1) | 3.0 (1.0) | 0.02** | 2.6 (1.1) | 2.9 (1.1) | 0.02* | 3.6 (1.0) | 4.3 (0.6) | 0.01 |
| Personal research ^d^ | 2.6 (1.1) | 2.7 (1.3) | 0.01 | 2.5 (1.1) | 2.3 (1.2) | 0.001 | 3.8 (1.1) | 4.0 (1.3) | 0.04 |
| Trust media coverage ^d^ | 3.0 (0.9) | 3.1 (0.9) | 0.002 | 3.0 (1.0) | 3.1 (0.9) | 0.001 | 4.1 (1.0) | 4.3 (0.9) | 0.001 |
| Advance thinking/planning ^d^ | 3.0 (1.3) | 3.2 (1.3) | 0.01 | 2.8 (1.3) | 3.3 (1.4) | 0.02 | 4.2 (0.8) | 4.6 (0.6) | 0.001 |
| Practical barriers ^e^ | 4.3 (0.8) | 4.4 (0.7) | 0.002 | 4.3 (0.8) | 4.3 (0.8) | 0.003 | 4.2 (0.8) | 4.6 (0.6) | 0.03* |
|  |  |  |  |  |  |  |  |  |  |
| P values and effect size (partial Eta squared) adjusted for child age and IMD2007 score. *: p<0.05, **: p<0.01, ***: p<0.001. Items collapsed to scales as follows: a: MMR beliefs, b: measles beliefs, c: social/parenting beliefs, d: information beliefs, e: standalone practicalities item | | | | | | | | | |
